# A 42‐year‐old female with sternoclavicular arthritis and breast abscess caused by brucellosis: A case report

**DOI:** 10.1002/ccr3.8071

**Published:** 2023-10-31

**Authors:** Mojtaba Varshochi, Reyhaneh Ravanbakhsh Gavgani, Fatemeh Ravanbakhsh Ghavghani, Sina Hamzehzadeh

**Affiliations:** ^1^ Department of Infectious Disease, Faculty of Medicine Tabriz University of Medical Sciences Tabriz Iran; ^2^ Department of Aquatic Biotechnology Artemia and Aquaculture Research Institute Urmia University Urmia Iran; ^3^ Liver and Gastrointestinal Disease Research center Tabriz University of Medical Sciences Tabriz Iran

**Keywords:** abscess, arthritis, brucellosis, sternoclavicular

## Abstract

**Key Clinical Message:**

The main target of this report was that brucellosis can occur in unexpected and very rare patterns. We reported this patient to acknowledge all of clinicians especially those living in the endemic areas, that these rare complications of brucellosis can also be treated by the standard treatment of this disease.

**Abstract:**

Brucellosis is a thousand‐face disease and common zoonotic infection in endemic regions. A 42‐year‐old female was admitted with sternoclavicular arthritis and breast abscess. After laboratory investigation and imaging, positive serological test results and positive blood culture for Brucella revealed acute sternoclavicular arthritis and breast abscess due to brucellosis.

## 1 BACKGROUND

Brucellosis is one of the most common zoonotic diseases worldwide, especially in Asia and Middle Eastern countries such as Iran.^1^, ^2^, ^3^ It is transmitted to humans through consumption of unpasteurized dairy products and direct contact with infected animal tissues.^4^, ^5^, ^6^ Brucellosis can involve any part of the body including the musculoskeletal system, genital organs, gastrointestinal system, nervous system, respiratory system, circulatory system, skin, heart, liver, spleen, bone, kidney, brain, epididymis, ovary, and gallbladder^7^, ^8^, ^9^ and it causes a systemic infection from asymptomatic disease to fatal illness^10^, ^11^ and it can mimic other infectious and noninfectious diseases.^4^, ^12^ So it is known as a “great imitator”.^10^

The onset of the disease can be insidious and when specific organ involvement is detected it is defined as focal brucellosis.^2^, ^10^, ^11^ In addition, arthritis, spondylitis, endocarditis, meningitis, and orchitis due to brucellosis are not uncommon^10^, ^11^, ^13^; but cutaneous and subcutaneous involvement with brucellosis are uncommon.^14^ Although soft tissue locations are usually related to a penetrating injury, the hematogenous spread is considered an important pathologic route.^8^, ^13^, ^15^

Although breast infection with granulomatous mastitis is described in animals, infection of mammary glands with Brucella is extremely rare in human brucellosis.^13^, ^14^, ^15^ Mammary glands infected with Brucella are reported in few cases and it is often hematogenous like a metastatic abscess in endocarditis.^11^ Gasser et al. reported the isolation of *Brucella melitensis* from a suspected breast tumor of a woman who also had uveitis.^13^

Osteoarticular lesions which are the most common complications in brucellosis include peripheral arthritis, osteomyelitis, spondylitis, sacroiliitis, and bursitis. Common joints consist of the knee, hip, ankle, and sacroiliac and less frequent ones include the shoulder, sternoclavicular, and temporomandibular.^10^, ^11^, ^16^, ^17^

## 2 CASE PRESENTATION

A 42‐year‐old Middle Eastern female patient from Iran was admitted to Sina Medical Research & Training Hospital on September 24, 2019 suffering from pain in the left sternoclavicular joint with spread to the left neck. She was a married woman with two children and was a hairdresser and tattoo artist. She had a history of left breast abscess one and a half month ago which was drained under an ultrasound guide. Five milliliter of concentrated pus were drained and sent to the culture, and the result was negative. She had no history of specific disease except a history of penicillin allergy and uterine myoma surgery 7 months before admission. She developed pain in the left sternoclavicular joint with spread to the left neck, shoulder and submental area 2 weeks before hospitalization, which gradually intensified and led to neck pain and limited movement of the left upper limb due to pain. Erythema and heat and swelling were added later to the sternoclavicular joint. The shoulder pain was reduced by using a slightly warm compress on it. She was hospitalized with a diagnosis of acute sternoclavicular joint arthritis owing to a lack of response to outpatient treatments and topical compounds. According to the abovementioned history and examination, the patient was admitted with the diagnosis of acute left sternoclavicular arthritis.

Over one and a half months, she had been taking co‐amoxiclav, dexamethasone, celecoxib, piroxicam, Gabapentin, vitamin B supplements, cephalexin, and vitamin E. She had no fever, chills, headache, nausea, and vomiting but she occasionally complained of scant purulent discharge from the left breast and a sense of heaviness in it. Examination of the heart, lungs, and abdomen were normal and she had no skin rash. Limited movement of the left upper limb was due to pain in the left sternoclavicular region and this region had slight erythema and tenderness in the palm. The right breast was normal and the left breast was tense.

Her vital signs at admission were as follows: blood pressure: 110/70, body temperature: 36.8 axillary, pulse rate: 82, and respiratory rate: 18. Cardiac echocardiography, chest and neck CT scan as well as liver and spleen ultrasound were normal. No abdominal and pelvic CT was done to rule out liver and spleen abscess.

We started to study the cause of acute sternoclavicular arthritis. MRI of the left sternoclavicular joint was performed, there was subarticular bone marrow edema at both the clavicular and sternal sides of the left sternoclavicular joint as well as some surrounding deep soft tissue edema, in favor of osteoarthritis. No obvious bony erosion was detected.

Ultrasound was conducted on the left breast because of a discharge from it and the result showed a fibrocystic change of both breasts and a hypoechoic mass in the left breast of 6.3 × 4.7 cm. A core needle biopsy of a left breast abscess was performed and the reported biopsy by the pathologist was as follows: breast tissue with moderate mixed inflammation, fibrocystic change, and foci of adenosis with florid hyperplasia. For definite diagnosis and ruling out of atypical florid hyperplasia immunohistochemistry (IHC) staining for P63, SMA,CK5/6, and ER were recommended. IHC findings did not confirm atypical changes.

Breast abscess culture revealed few colonies of coagulase‐negative staphylococcus. Then, blood culture was done for the patient and the results showed a bacteremia of *Brucella melitensis*. Serology of brucellosis was also positive (Wright: 1/160).

Consequently, the patient was treated with Streptomycin and doxycycline. Four days later she felt improvement in the left sternoclavicular joint. Pain and tenderness in the joint significantly decreased and the patient was able to move the left upper limb and the discharge from the patient's left breast was stopped. After the diagnosis of brucellosis and good response to treatment, on the 14th day of hospitalization, the patient was discharged and requested for continuing treatment order with doxycycline and Streptomycin for 1 week followed by doxycycline and rifampin and following up on outpatient basis. Total duration of the patients treatment lasted 30 days.

The laboratory workup is summarized in Table 1.

**TABLE 1:** Laboratory tests during hospitalization.

Parameter	Value
White blood cells	9.9 × 103/μL (Neutrophils =68.2%, Lymphocytes =27.5%)
Hemoglobin	11.4 g/dL
Platelets	250,000 /mm^3^
Prothrombin time	13.8
Partial thromboplastin time	35
INR	1.2
Urea	26 mg/dL
Creatinine	0.8 mg/dL
Blood Sugar	86
Na	138 mg/dL
K	4.5 mg/dL
Calcium	Total = 8.1 mg/dL; Ionized = 1 mg/dL
Phosphorus	3.2 mg/dL
Albumin	4.3 g/dL
Uric acid	0.2 mg/dL
Aspartate aminotransferase	17 IU/L
Alanine aminotransferase	24 IU/L
Alkaline phosphatase	232 IU/L
Total bilirubin	0.34 mg/dL
HIV‐Ab	Negative
Creatine kinase (CK)	37 U/L
Antinuclear antibodies (ANA)	Negative
Rheumatoid factor (RF)	Negative
Anti‐cyclic citrullinated (Anti CCP)	Negative
Erythrocytes sedimentation rate (ESR)	53 mm/h
C‐reactive protein (CRP)	36 mg/dL
Lactate dehydrogenase	299 IU/L
Wright	1/160
Coombs Wright	1/320
2 mercaptoethanol (2ME)	1/80
Blood Culture× 2	Brucella was isolated on Castaneda medium after 7 days
Breast abscess culture	Few colonies of coagulase negative staphylococcus
Urine analysis	Normal
Urine culture	Negative

Abbreviations: HIV Ab, Human immunodeficiency virus‐antibody; INR, international normalized ratio.

A follow‐up was done for the patient 30 days after discharge, she had been in good clinical condition with normal sternoclavicular joint, and the examination of her left breast was normal and without any discharge. Also, additional ultrasound was done for her breasts and it showed a fibrocystic change in both breasts without either evidence of collection or axillary lymph nodes. No laboratory tests including Brucella titer was done in the follow up.

## 3 DISCUSSION

In this article, we reported a 42‐year‐old woman who had no history of livestock contact, sternoclavicular joint or left breast trauma, and also there were not any risk factors of sternoclavicular arthritis such as intravenous drug use, distant site of infection, diabetes mellitus, trauma, and infected central venous line. She had experienced purulent discharge from the left breast and underwent left breast abscess drainage and antibiotic treatment on an outpatient basis one and a half months before hospitalization, but there was no improvement so occasional discharge continued following abscess evacuation. Furthermore, 2 weeks before admission, pain and redness, and swelling of the left sternoclavicular joint were added. So she was hospitalized with the diagnosis of acute left sternoclavicular arthritis for further examination. Studies suggest that breast abscess is most likely due to hematogenous spread.^12^ MRI showed signs of left sternoclavicular arthritis. In addition, laboratory tests that were performed to rule out causes of arthritis, including rheumatoid arthritis and systemic lupus erythematosus, all were negative.^2^ Since brucellosis is one of the causes of arthritis in endemic areas, blood culture and serology of brucellosis were requested. Blood culture × 2 was reported positive for brucellosis and Wright agglutination test:1/160, Coombs Wright test: 1/320, 2‐mercaptoethanol (2ME): 1/80 in favor of brucellosis.^5^, ^10^, ^11^ According to the patient's complaint about purulent discharge from the left breast and history of abscess drainage one and a half months ago, at the same time as we were performing diagnostic tests for left sternoclavicular arthritis, a breasts ultrasound was performed which showed a fibrocystic change of both breasts and a mass in the left breast then she underwent left breast abscess biopsy. The results of pathology and IHC were not in favor of malignancy. Breast abscess culture was reported as having few colonies of coagulase‐negative staphylococcus which is considered as contamination because she did not have a fever, leukocytosis and other systemic signs also colony count was low.

Breast abscess due to brucellosis is extremely rare. So to rule out endocarditis and its metastatic abscess^11^ to the breast and a sternoclavicular joint, transthoracic echocardiography was performed and its result was normal, without any vegetation or valve abnormality.

Fever, chills, sweating, fatigue, headache, splenomegaly, hepatomegaly, arthralgia and musculoskeletal pain, which are seen in most patients with generalized and localized brucellosis,^1^, ^5^, ^11^ were not present in our patient and she also had no other positive findings except left sternoclavicular arthritis and left breast abscess.

As reported in other studies, there were no specific hematological or biochemical findings in our case.^2^, ^11^, ^12^ There was no leukocytosis in this patient and liver function tests were normal but ESR and CRP were high.

As Since the diagnosis of brucellosis arthritis is confirmed by signs and symptoms of arthritis (pain, tenderness, swelling of the joint)^17^, ^18^ in the presence of an antibody titer greater than 1:160 in the tube agglutination test or by a positive culture,^10^, ^11^ brucellosis with the subtype of melitensis was diagnosed in our patient and considering two very rare complications of brucellosis: left sternoclavicular arthritis and left bursal abscess both simultaneously in this patient due to the lack of history of trauma and external inoculation and because she had no history of direct contact with livestock, we concluded that these complications are hematogenous. Therefore, with the diagnosis of localized brucellosis, which requires a combination of antibiotics and a prolonged course of treatment to prevent failure or relapse of brucellosis,^2^, ^10^, ^11^ antibiogram was done for the patient and according to its results, Brucella was sensitive to streptomycin and doxycycline. Therefore the patient treated with a standard regimen (streptomycin and doxycycline for 2 weeks, then rifampin and doxycycline for 10 weeks). On the fourth day of treatment, she responded and the joint symptoms improved significantly the discharge from the patient's left breast was stopped after starting the treatment and she was discharged and recommended to continue the outpatient treatment and follow‐up. In a case report by Al Abdely et al. a patient had a breast abscess that was confirmed to be Brucella. Their patient was treated with doxycycline and trimethoprim/sulfamethoxazole and showed an immediate improvement.^11^ In our report it was the same. Therefore, because of this dramatic response to the therapy, we concluded that the organism responsible for the arthritis and abscess of this patient was also probably *Brucella melitensis*. The prognosis of Brucella arthritis will be very good if appropriate and in time treatment is begun.^12^, ^19^ Unlike spondylitis, both sacroiliitis and peripheral arthritis are nondestructive and quickly curable with no sequelae.^2^, ^12^

Thirty days after discharge at the follow‐up visit, the patient did not relate a feeling of stiffness or heaviness in the left breast and occasional discharge from it which she had previously experienced, and also left sternoclavicular arthritis completely was cured. She completed treatment of two localized complications of brucellosis simultaneously (sternoclavicular arthritis and left breast abscess) without any complications.

## 4 CONCLUSION

Each of the complications of sternoclavicular arthritis and brest abscess due to brucellosis are extremely rare. However, we found both of these complications simultaneously in one patient. These complications can be successfully treated using the standard treatment of brucellosis (streptomycin and doxycycline for 2 weeks followed by rifampin and doxycycline for 10 weeks). Given that brucellosis is a thousand‐face disease, especially in endemic areas, clinicians in all regions of the world, especially endemic areas should be familiar with rare complications of it so we reported this patient with very rare complications of this zoonotic disease and its successful treatment.

## AUTHOR CONTRIBUTIONS

**Mojtaba Varshochi:** Data curation; formal analysis. **Reyhaneh Ravanbakhsh Gavgani:** Conceptualization; data curation; project administration; writing – original draft. **Fatemeh Ravanbakhsh Ghavghani:** Conceptualization; data curation; investigation; methodology; project administration; resources; writing – original draft. **Sina Hamzehzadeh:** Formal analysis; investigation; project administration; software; supervision; validation; visualization; writing – review and editing.

## CONFLICT OF INTEREST STATEMENT

The authors declare that they have no conflict of interest.

## ETHICAL APPROVAL AND CONSENT TO PARTICIPATE

Ethics approval code: IR.TBZMED.REC.1399.1024.

## CONSENT

Written informed consent was obtained from the patient for publication of this case report and any accompanying images. A copy of the written consent is available for review by the Editor‐in‐Chief of this journal.

## Data Availability

The data that support the findings of this study are available from the corresponding author upon reasonable request.
